# Creative Cities Creating Connections: Fostering Cross-Age Interaction Through Leisure

**DOI:** 10.1093/geroni/igaa057.1416

**Published:** 2020-12-16

**Authors:** Rachel Douglas, Anne Barrett

**Affiliations:** Florida State University, Tallahassee, Florida, United States

## Abstract

Older and younger people interact infrequently in most social realms – a pattern stemming from institutional, cultural, and spatial age segregation. Increasing cross-age interaction offers promise as a strategy for not only enhancing social connections but also reducing ageist attitudes. We argue that a social realm with untapped potential for creating these connections is leisure, particularly within creative cities. To explore this possibility, our study examines cross-age interactions in Key West, Florida – a leisure-oriented city that promotes creativity through its social and built environment. Using participant-observation and interview data (n=126) from 2017 to 2019, we examined leisure experiences of tourists and residents, aged 23 to 83. Findings indicate that creative cultural contexts can counter ageist attitudes by promoting cross-age interaction. Data analysis revealed three processes encouraging these shifts – promoting diversity and acceptance, enhancing older adults’ participation, and nurturing intergenerational bonds. Ongoing cross-age interactions were fostered through the island’s welcoming philosophy and encouragement of out-group acceptance and diversity. They were further fostered by the island’s wide array of leisure activities and proximity of shared spaces that enabled older adults to readily pursue leisure with younger people. Cross-age ties also were promoted by the island’s focus on family-friendly leisure experiences. Our research highlights the potential of creative cities to promote age integration, and reduce ageism, through its cultural practices within leisure spaces.

